# Cognitive abilities predict naturalistic speech length in older adults

**DOI:** 10.1038/s41598-024-82144-w

**Published:** 2024-12-28

**Authors:** Patrick Neff, Burcu Demiray, Mike Martin, Christina Röcke

**Affiliations:** 1 Department of Otorhinolaryngology, Head and Neck Surgery, University Hospital Zurich, University of Zurich, Zurich, Switzerland; 2 Department of Psychiatry and Psychotherapy, University of Regensburg, Regensburg, Germany; 3 Healthy Longevity Center, University of Zurich, Zurich, Switzerland; 4 Department of Psychology, University of Zurich, Zurich, Switzerland

**Keywords:** Cognitive ability, Real-life speech, Language production, Social activity, Ambulatory assessment, Psychology, Human behaviour

## Abstract

Past research has demonstrated the association between social engagement and the maintenance of cognitive abilities. However, inconsistent definitions of social engagement have posed challenges to systematically investigate this association. This paper addresses the role of social relationships in cognitive functioning among older adults, focusing on the real-life communication indicator—length of own speech—as a measure of social activity. Utilizing advanced technology to unobtrusively measure older adults’ real-life speech, this study investigates its association with various cognitive abilities and sociodemographic factors. Differential cognitive measures, and sociodemographic data including factors like age, sex, education, income, persons living in the same household, loneliness, and subjective hearing status were included. Audio data of 83 participants are analyzed with a machine learning speaker identification algorithm. Using Elastic Net regularized regression, results indicate that higher levels of working memory, cognitive speed, and semantic fluency predict own speech in everyday life. While having no partner negatively predicted own speech length, we unexpectedly found that higher hearing status was related to lower speech frequency. Age was neither a relevant predictor in the regression nor correlated with any other variables. We discuss implications and future research applications based on the findings from our novel approach.

## Introduction

The United Nations has declared the current decade (2021–2030) as the “Decade of Healthy Aging”. At the center of this global initiative is the healthy aging model from the World Health Organization (2015). This model conceptualizes healthy aging as the process of developing and maintaining the functional ability that enables well-being in old age. One important aspect of functional ability is the ability to build and maintain social relationships in everyday life. Social relationships have attracted much interest in aging research: Social engagement across the lifespan is found to be associated with the maintenance of cognitive abilities and with reduced risk for cognitive impairment, including dementia and Alzheimer’s Disease^1–6^.

An important challenge in trying to understand the effect of social relationships on cognitive function is the use of unclear and divergent definitions of different social factors in the literature^7,8^. Berkman and colleagues^9^ claimed that researchers used these terms loosely and interchangeably (e.g., social networks, social ties, social integration, social support) and, thus, aimed to clarify terms using a single framework. They focused on social relationships as including three different aspects: social networks, activities and support. Social networks are “the web of social relationships that surrounds an individual” (p. 847) with investigated characteristics, such as network size, relationships between network members, frequency of contact between network members^9^. Social activities are known as social participation (e.g., meeting friends, attending events, volunteering) which have been extensively studied in activity participation research^2,10^. Finally, social support refers to one’s perception of the availability of help or support from those in their social network (e.g.,^11,12^). In two reviews, Kuiper and colleagues^3,8^ explain that social networks and social activities represent the “structural” aspects of social relationships, whereas social support represents the “functional” aspects of social relationships. Research investigating the impact of social relationships on cognitive functioning most commonly focuses on the frequency of social activity participation^10,13^, followed by social network characteristics^14,15^ and social support (e.g.,^7,16^).

Where do verbal interactions or conversations with other people stand in this framework? They seem to be most fitting with the “social activity” dimension, which includes meeting and interacting with other people. How can researchers measure and use speech/language production as a distinct aspect of social activities? Zuelsdorff and colleagues^17^ have developed a “verbal interaction” questionnaire in which participants are asked to estimate and report the quantity of their verbal interactions in eight social domains (i.e., spouse/partner, other family, friends, colleagues, club/hobbies, religious meeting attendance, interactions with strangers, and “other” category). Participants respond, based on time per day or time per week depending on the domain in question, on a six-point scale ranging from “none” to “more than two hours”. This type of self-report method has been used extensively in the literature on social activity participation, where participants are asked how often they have participated in an activity in a period of weeks, months, or years (e.g.,^10,18–20^). In spite of its acknowledged psychometric value and practical efficiency, the self-report method may show measurement limitations and biases, such as recall bias, response styles, demand characteristics, social desirability, limitations to introspection and research participant burden and capacity (e.g.,^21^).

Real-life language production of older adults has been investigated in very few studies (e.g.,^22^), which used the Electronically Activated Recorder (EAR;^23^) to examine the linguistic complexity of speech across various social contexts^22,24,25^. These studies have created verbatim transcripts of participants’ real-life speech and conducted text analytics on the transcripts. Their results showed differences in vocabulary richness and grammatical complexity across different age groups (i.e., young versus old), social contexts (e.g., working versus socializing) and interlocutors (e.g., spouse versus strangers). None of these studies, however, included cognitive ability measures or explored the relation between real-life language production and cognitive abilities to further explore the language-cognition interplay. To our knowledge, there are only two studies that have examined this link using naturalistic language collected by the EAR: Polsinelli and colleagues^26^ have examined the relation between patterns of daily word use and executive functioning (working memory, shifting and inhibitory control) in healthy older adults. The verbatim transcripts of participants’ real-life speech were text-analyzed using the Linguistic Inquiry and Word Count^27^. They found that, controlling for age, education, and gender, higher levels of executive functioning (particularly working memory) were associated with more analytic (e.g., more articles and prepositions), complex (e.g., longer words), and specific (e.g., more numbers) language use. Ferrario and colleagues^28^ have worked on the same dataset with a machine learning approach to predict working memory using linguistic measures (i.e., grammatical complexity, vocabulary richness), part-of-speech tags (with natural language processing) and social context information. They showed that machine learning and NLP may support the prediction of working memory using linguistic measures and social context information. In summary, these two studies have examined the relation between the linguistic qualities of naturalistic speech and cognitive abilities without focusing on the social aspects of language use, however.

Findings on the association between social activity and cognitive abilities are not always clear due to the different operational definitions, study designs and methodologies used across studies. In their systematic review of the literature, Kelly and colleagues^7^ have shown a positive relationship between social activity participation and global cognition, and more specifically executive functioning, working memory, visuospatial abilities and processing speed. There were no significant relations with episodic memory, verbal fluency, reasoning or attention^7^. Zuelsdorff and colleagues^29^ examined older adults’ quantity of verbal interactions via a self-report measure and found a positive relation with verbal learning and memory, but not with working memory or processing speed (controlling for lifestyle covariates). In sum, one can conclude that different aspects of social relationships seem to have different effects on specific cognitive processes (e.g.,^7,30^).

### The current study

As mentioned above, there are very few studies that have examined older adults’ verbal interactions in the real world^31,32^. The two studies that have studied the relation between their naturalistic speech and cognitive abilities have not focused on speech as an indicator of social activity^26,28^. Furthermore, these studies have relied on the manual transcription of all naturalistic speech produced by participants, which is an extremely time-consuming and thus costly endeavor. Hence, the current paper, for the first time, measures the length of participants’ naturalistic speech and conceptualizes it as a simple and objective indicator of social activity as it occurs in daily life. Unobtrusive audio sampling in real-life settings (i.e., EAR method) is now commonly being integrated into modern ambulatory assessment studies with a wide range of mobile sensing methods^33^, as well as different mobile devices. In our study, we have implemented audio sampling into the uTrail, our custom-made, small clip-on activity tracking device that also collects GPS and accelerometry data^34^. The basic social activity indicator made possible by this method could provide researchers with an efficient tool to quantify social activities in daily life.

We examined the widely studied, important link between social activity and cognitive abilities with this novel approach. Due to the exploratory nature of the study that aims to describe relationships, we did not formulate specific hypotheses. Our main research goals were to (1) objectively and unobtrusively observe older adults’ real-life conversations (via naturalistic observation) and to acquire an estimate of the length of their own speech as an indicator of their social activity; (2) explore whether and how the length of real-life speech is associated with key aspects of cognitive ability (i.e., working memory, processing speed, executive functioning, episodic memory, verbal knowledge, verbal phonemic fluency, verbal semantic fluency); and (3) explore whether and how the length of real-life speech is associated with sociodemographic variables (e.g., civil or partner status) or the subjective hearing status.

## Materials and methods

### Transparency and openness

In accordance with local ethical guidelines and data privacy laws, de-identified data and/or materials from this study cannot be made accessible to the public. The primary reason for this is the presence of personal data within the audio recordings, which include not only the participants’ speech, but also the voices of third parties who may not have consented to have their conversations recorded and analyzed. This risk extends even to de-identified data, as voice patterns and context could potentially allow for identification of single individuals. Additionally, participants were not asked for, nor did they provide, consent for their data to be shared broadly beyond the scope of this study. They only consented to their data being presented at the within-person level in terms of visualizations to the general public and for use in research-related publications. We are committed to respecting the privacy and confidentiality of all individuals involved in the study. Summary statistics of participants’ length of own speech can be made available upon reasonable request to the first author. The analytic code for this study is not available for two main reasons. Firstly, without access to the specific dataset used in this research, the utility of the analytic code is severely limited. Secondly, the methodologies and analytical procedures applied are comprehensively described within the paper, offering sufficient information for understanding the conducted analyses. Neither the study design and hypotheses nor the analytic plan of this study have been pre-registered.

### General information

We pursued our research goals using data from a large interdisciplinary ambulatory assessment project on mobility, activity, and social interactions of older adults^34^. The study procedures followed agreements in the Declaration of Helsinki and were approved by the Ethics Committee of the Faculty of Arts and Social Sciences of the University of Zurich (permission no. 17.2.4). All participants provided written informed consent.

### Participants

We recruited participants through the lead institute’s survey center. Inclusion criteria were age 65 years or above, computer and internet access at home, sufficient vision to use the smartphone, and a score of 27 or above in the Mini-Mental State Examination. A total of 150 older adults fitted the study criteria and were compensated with 200 Swiss Francs. For a detailed overview of our power analyses to determine sample size, please see the study protocol^34^. All participants spoke Swiss German as their primary language. The analyses discussed in this paper utilized a subset of participants for whom we had comprehensive ground truth audio data from the lab and complete baseline cognitive and sociodemographic information. The ground truth audio data comprised a short audio recording in a silent room at our laboratories with no distracting sounds or voices in the background. This clean audio file served the machine learning speaker identification algorithm (described below) to clearly identify and extract the voice streams of the targeted participants. As a result, statistical analyses were conducted on a revised total of 83 participants (see details below for more information).

### Study design and procedures

Participants completed questionnaires on sociodemographic information and health, as well as psychometric tests measuring cognitive performance at baseline. Questionnaires were assessed online using SoSci Survey^35^ and cognitive tests performed at our laboratories as part of the data collection of our large-scale study^34^. No changes to the questionnaire or scaling of the questionnaires via other platforms were performed. Across four weeks, participants carried a custom-built mobile sensor (the “uTrail”) with them during their waking hours as they went about their daily lives. Participants did not interact with the device at any time and sensor recordings, including audio recordings (see below), were triggered automatically.

### Audio recorder

The uTrail device, measuring 5.5 cm in diameter, 2 cm in depth, and weighing less than 100 g, is equipped with a clip that allows it to be securely attached to the waist, either on a belt or waistband. It includes a microelectro-mechanical systems (MEMS) microphone (Model: NVENSENSE INMP510ACEZ-R7), enabling audio recordings comparable to those of the Electronically Activated Recorder (EAR;^23^). The audio recordings consisted of 50-s ambient sound snippets that were sampled every 18 min as a sensor-based indicator of speech and social interactions in daily life. A VS1063 audio MP3 encoder (VLSI Solution Oy, Finland) was used with a sample rate of 48 kHz and an encoding rate of 160 kbps (Codec: MPEG Audio layer 1/2). The recorded mono MP3 files were stored on a removable 8 GB SD card, using the FAT file system.

### Measures

#### Cognitive variables

Overall *cognitive status* was screened using the Mini-Mental-Status-Examination (MMSE;^36^). *Verbal knowledge* was measured using the Mehrfachwahl-Wortschatz-Intelligenztest (Multiple Choice-Vocabulary-Intelligence Test;^37^) in which participants are presented with 37 short word lists consisting of one word and several non-word distractors and have to identify the real word. *Verbal fluency* was measured using both a test for phonemic fluency (Leistungsprüfsystem [LPS6];^38^) and one for semantic fluency (Regensburger Wortflüssigkeitstest [RWT];^39^). *Processing speed* was assessed with Digit Symbol Test from the German version of the Hamburg-Wechsler Intelligence Test for Adults (HAWIE);^40^) and performance on the Trail Making Test-A^41^. *Working memory* was assessed by the repeated numbers task forward and backwards from the HAWIE^40^. We measured *episodic memory* with the Verbal Learning and Memory Test (VLMT,^42^), and *executive functioning* with the task switching component of the Trail Making Test, using performance on the TMT-B and the difference score between Test Version A and B (TMT;^41^).

#### Sociodemographic variables and hearing status

We accounted for several sociodemographic variables including *age* in years, *sex* (0 = women, 1 = men), *years of education, monthly income* (1 = up to 3′000 Fr., 2 = 3′001 to 4′000 Fr., 3 = 4′001 to 6′000 Fr., 4 = 6′001 to 8′000 Fr., 5 = 8′001 to 12′000 Fr., 6 = more than 12′000 Fr.), *civil or partner status* (0 = single, divorced, widowed, 1 = married or in long-term relationship), and *number of persons living in same household.* Participants also reported their *subjective hearing status* using the item “How would you rate your current hearing (with a possible hearing aid)?” on a scale from 1 (deficient) to 5 (excellent). Social isolation or loneliness was assessed by the UCLA Social Loneliness Scale^43^.

#### Own speech: speaker identification algorithm

The extraction of the frequency of each participant’s real-life speech from the uTrail EAR audio data was the key methodological task of this study. We employed the software Vocalise^44^ on a virtual machine using Windows 10 operating system. This software implements x-vector Probabilistic Linear Discriminant Analysis (PLDA) to accurately identify specific speakers in audio recordings. We referenced the algorithm to an initial clean audio recording taken from the target speaker in the lab at study baseline (i.e., ground truth). From an ethical and data safety standpoint, the application of the speaker identification algorithm (here: Vocalise) is crucial as it ensures that only relevant segments containing the target speaker are identified and used in subsequent analyses. The temporal granularity of the Vocalise analysis is 5 s (current limitation of the software). The derived measure of the length of a participant’s own speech is calculated as the ratio of the cumulative length of the identified speech snippets to the total duration of the audio recordings in seconds. To tailor the Vocalise algorithm to our specific needs, the algorithm has been specifically optimized for our data configuration in several iterations with the manufacturer.

From the original 150 participants enrolled in the study, 87 participants recorded a lab audio file at study baseline used as the ground truth for the Vocalise algorithm. This subsetting of the study sample was necessary, as the Vocalise algorithm is dependent on clean ground truth data, especially in the case of our complex and noisy real life audio data involving Swiss German from the audio recorder. The ground truth recording procedure was not part of the initial study design and was introduced only after the Vocalise algorithm’s analysis strategy was established, which resulted in the reduced final sample size.

### Data analyses

Analyses were performed using R^45^. The data manipulation and visualization were performed with the tidyverse package^46^. For the machine learning elastic net regression task, we used the caret package^47^. The network analysis and visualization were conducted with the qgraph package^48^ and the correlation matrix plot with the corrplot package^49^. Due to systematically missing values in the cognitive ability and sociodemographic data, we had to exclude four cases from the statistical analyses. Given the pattern of missing values, imputation methods were deemed unfeasible. Consequently, we refrained from using these four cases for any further analysis. The final, total number of participants analyzed and reported was reduced to 83.

#### Elastic Net regression

In our primary analysis of the relations between own speech length, cognitive ability and sociodemographic variables, we employed an Elastic Net regression model, a machine-learning statistical technique that combines the strengths of Ridge and Lasso regression^5^. This hybrid approach allows for the handling of multicollinearity between predictors and the selection of the most relevant features for prediction. The Elastic Net model uses two hyperparameters, alpha and lambda. The alpha parameter controls the balance between Ridge (alpha = 0) and Lasso (alpha = 1) regularization, allowing for a compromise between the two. In our study, the optimal alpha was determined through cross-validation (see below). The lambda parameter, on the other hand, controls the overall strength of the penalty, with larger values leading to stronger regularization. This parameter was also selected using cross-validation to avoid overfitting and to ensure that our model generalizes well to new data.

Prior to model building, categorical variables were dummy coded (i.e., sex and partner status) and data was preprocessed using centering and scaling to ensure that all variables were on a comparable scale. This step is crucial for the Elastic Net approach, as it standardizes the predictors, making the penalty term fair and the model more robust. Elastic Net does not require predictors or residuals to follow a normal distribution. The dataset was then partitioned into training and test sets in a 70:30 ratio. For training, tenfold cross-validation was repeated ten times to validate model performance. We performed a grid search on the Elastic Net model over ten alpha values (from 0 to 1) and one hundred lambda values (from 0.00001 to 0.1). Alpha represents the penalty type, blending Lasso (alpha = 1) and Ridge (alpha = 0) penalties, while lambda controls the degree of shrinkage. The hyperparameter combination offering the best cross-validated performance was chosen, ensuring an optimal model balance between bias and variance.

Model performance was assessed using Root Mean Square Error (RMSE) and R-squared measures. RMSE provides an estimate of the standard deviation of the residuals, giving us an understanding of the spread of the unexplained variance. R-squared, on the other hand, represents the proportion of variance in the dependent variable that can be explained by the predictors in the model. The importance of each variable in the model was then determined by calculating the absolute value of the standardized coefficients. This process considers both the size of the variable’s effect (the coefficient) and the scale of the variable (since the coefficients are standardized) ensuring that the importance rankings are not unduly influenced by the scale of the variable, reflecting the genuine contribution of each predictor to the model.

#### Correlation network analysis

A secondary, explorative network analysis was conducted using the qgraph package in R, applying Spearman correlation due to non-normal distribution of some of the predictor variables to identify relationships between variables and clusters thereof. The resultant correlation matrix served as the foundation for constructing the network graph. The “spring” layout of qgraph was used employing a force-directed algorithm that aims to position highly correlated nodes closer together and less correlated nodes further apart, thereby visually enhancing the strength of the relationships between variables. This enables a more intuitive understanding of the structure of relationships within the dataset and, alongside with the partial correlation matrix (see below), is intended to provide an exploratory insight into variable dynamics which are not evident from the main Elastic Net regression model due to the absence of bivariate correlations of the predictors and the coefficient penalization inherent to the algorithm (i.e., setting non-relevant coefficients to zero). P-value thresholds were set to p < 0.05 and the statistical search (sub)space was FDR-corrected; the correlations used are the raw correlations between variables with correlation strength coded in edge thickness. The color-coded edges in the graph represent positive (green) and negative (yellow) correlations between nodes (variables). Significant edges (p < 0.05, FDR-corrected) are coded in solid lines, non-significant edges with dashed lines.

#### Partial correlation analysis

In addition, a partial correlation (Spearman’s) matrix is presented for further exploration of bivariate correlations controlling for all other correlations of the bivariate pairs. Significance level was set to p < 0.05 and FDR correction for multiple comparisons was applied.

## Results

### Descriptive statistics

**Fig. 1:**
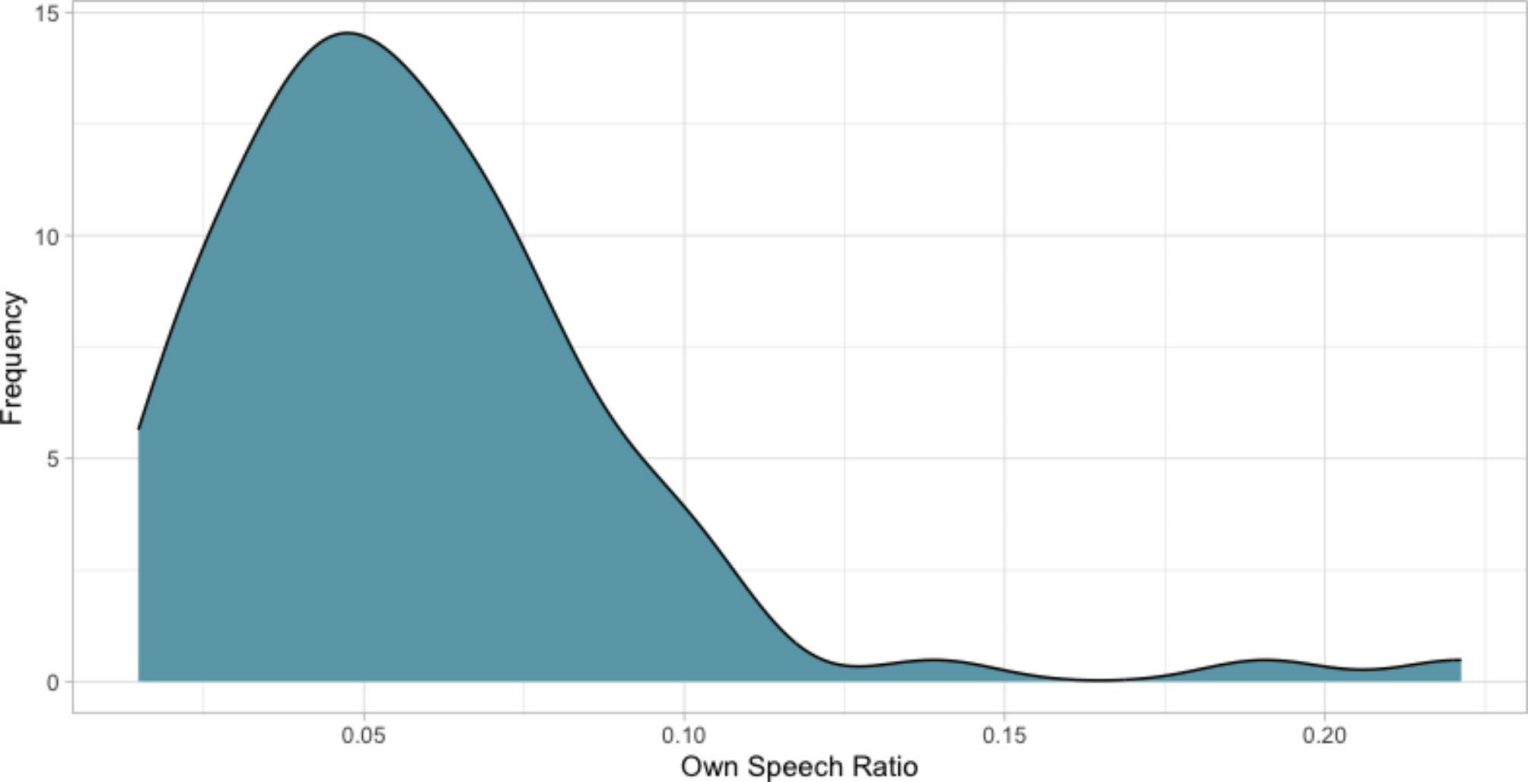
Distribution of length of own speech (dependent variable, ratio 0–1). Mean = 6%, SD = 3% of total audio recording time.

Table 1 lists participants’ characteristics including all predictors and the dependent variable of the regression model. Figure 1 depicts the distribution of the target measure and dependent variable of the regression model, own speech ratio. The average ratio of own speech in all audio recordings is 6% (SD = 3%, Min = 1%, Max = 2%). 36 out of 83 participants in our final sample were male, and 51 reported to live in a partnership (i.e., either in a long-term relationship, married, or registered partnership).

**Table 1:** Participants’ characteristics.

Variable	Mean	SD	Median	Min	Max
Age (years)	73.46	5.89	72	65	91
Income *	3.08	1.44	3	1	6
Education (years)	14.48	3.36	14	8	24
# of persons in household	0.76	0.79	1	0	5
Hearing status	3.34	0.90	3	1	5
VLMT_number	46.70	9.57	46	24	67
TMT_ratio	2.60	0.84	2.46	1.24	4.79
TMT_A	42.07	14.99	38	23.55	96.15
TMT_B	106.20	46.46	96	46.1	352.4
MMSE	28.63	1.16	29	26	30
HAWIE_digit	40.30	6.30	40	22	56
LPS_score	36.25	7.84	36	19	55
HAWIE_memo	15.16	3.14	15	9	24
MWT_number	32.47	3.41	33	21	52
RTW_score	32.52	9.34	31	6	65
Own speech [%]	0.06	0.03	0.06	0.01	0.22
# audio files	1247.64	312.06	1344	235	1730

*Monthly income in Swiss Francs: 1 = up to 3′000 Fr., 2 = 3′001 to 4′000 Fr., 3 = 4′001 to 6′000 Fr., 4 = 6′001 to 8′000 Fr., 5 = 8′001 to 12′000 Fr., 6 = more than 12′000 Fr. # audio files = number of audio files recorded from the participant. HAWIE_digit = Hamburg-Wechsler Intelligence Test for Adults digit-symbol test. HAWIE_memo = Hamburg-Wechsler Intelligence Test for Adults repeated numbers task. LPS_score = Leistungsprüfsystem score (phonemic fluency). MMSE = Mini-Mental State Examination. MWT_number = Multiple Choice-Vocabulary-Intelligence Test, number correct (verbal abilities, semantic memory). RTW_score = Regensburger Wortflüssigkeitstest, score (semantic fluency). TMT_A = Trail Making Test Part A (numeric). TMT_B = Trail Making Test Part B (alphanumeric). TMT_r = Trail Making Test Ratio (B/A). VLMT_number = Verbal Learning and Memory Test, episodic memory.

### Elastic Net regression

**Fig. 2:**
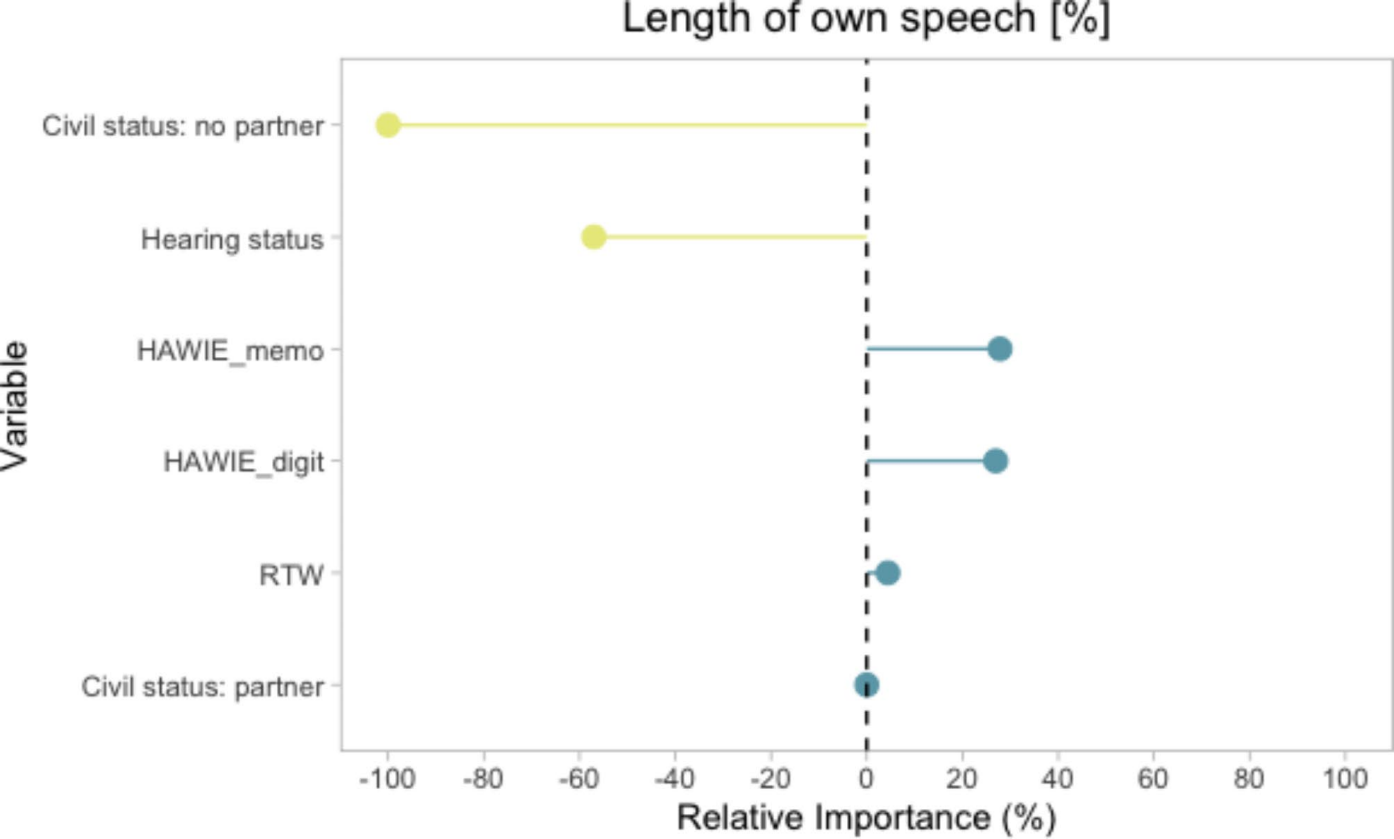
Variable importance in the elastic net model predicting length of own speech in real-life audio data. HAWIE_digit = Hamburg-Wechsler Intelligence Test for Adults digit-symbol test. HAWIE_memo = Hamburg-Wechsler Intelligence Test for Adults repeated numbers task. RTW = Regensburger Wortflüssigkeitstest (semantic fluency).

For primary analysis, we used an Elastic Net regression model to analyze the relationship between the response variable “own speech ratio” and the various predictor variables. The optimal hyperparameters, found through tenfold cross-validation, were alpha = 1 and lambda = 0.1. The final model achieved an RMSE of 1.361 and an R-squared of 0.189. The relevant predictors, sorted by the absolute values of their coefficients and their variable importance, can be found in Table 2 and Fig. 2, respectively. Overall, cognitive variables exerted less influence on the amount of own real-life speech than the no partner status or the subjective hearing status. The ‘no partner’ status contributed 100% of relative variable importance of the model (no partner related to less own speech), followed by hearing status with 57% (good hearing related to less own speech). Working memory (HAWIE_memo) and processing speed (HAWIE_digit) contributed 28% and 27%, respectively, with higher scores related to more own speech. Semantic fluency (RWT) accounted for 4% and a positive ‘partner status’ for < 1%, both predicting a higher amount of own speech. The results of this main analysis are comprehensively discussed in the Discussion section.

**Table 2:** Standardized coefficients of the Elastic Net model.

Predictor variable	Standardized coefficient
Civil status: no partner	-0.257
Hearing status	-0.147
Working memory (HAWIE)	0.072
Processing speed (HAWIE)	0.069
Semantic fluency (RTW)	0.014
Civil status: partner	< 0.001

### Correlation network analysis

**Fig. 3:**
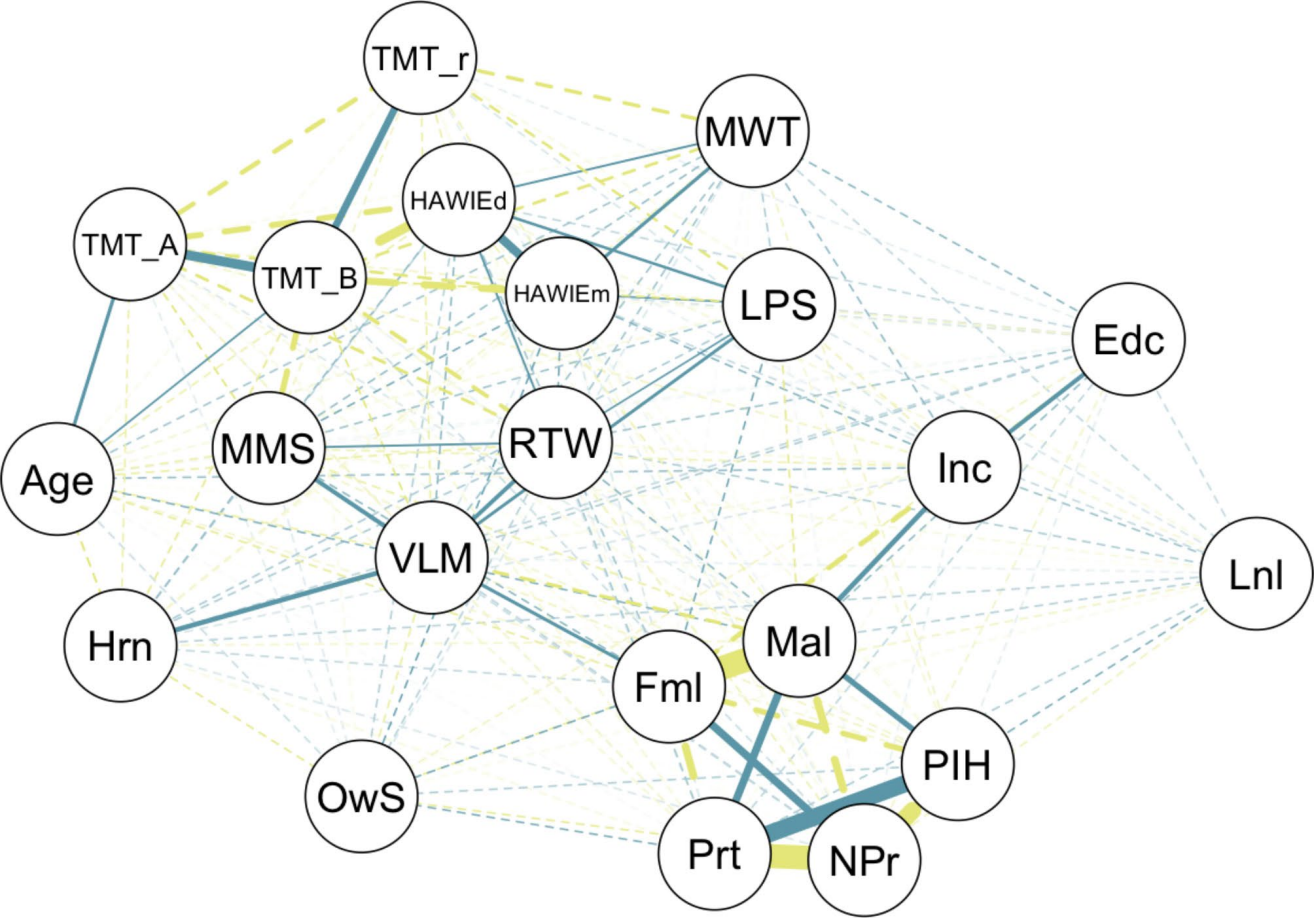
Network correlation cluster plot of the model’s variable set (force-directed graph, Spearman correlations). Positive correlations are coded in dark green, negative correlations in light green/yellow. Dashed lines indicate non-significant correlations, solid lines significant correlations (FDR-adjusted for multiple comparisons). Edc = Education. Fml = Female. HAWIEd = HAWIE_digit (Hamburg-Wechsler Intelligence Test for Adults digit-symbol test). HAWIEm = HAWIE_memo (Hamburg-Wechsler Intelligence Test for Adults repeated numbers task). Hrn = Hearing status. Inc = Income. Lnl = Loneliness. LPS = Leistungsprüfsystem (phonemic fluency). Mal = Male. MMS = MMSE (Mini-Mental State Examination). NPr = No partner. MWT = Multiple Choice-Vocabulary-Intelligence Test (verbal abilities, semantic memory). OwS = Length of own speech. Prt = Partner. PIH = Number of persons in household. RTW = Regensburger Wortflüssigkeitstest (semantic fluency). TMT_A = Trail Making Test Part A (numeric). TMT_B = Trail Making Test Part B (alphanumeric). TMT_r = Trail Making Test Ratio (B/A). VLM = VLMT_number (Verbal Learning and Memory Test, episodic memory).

**Fig. 4:**
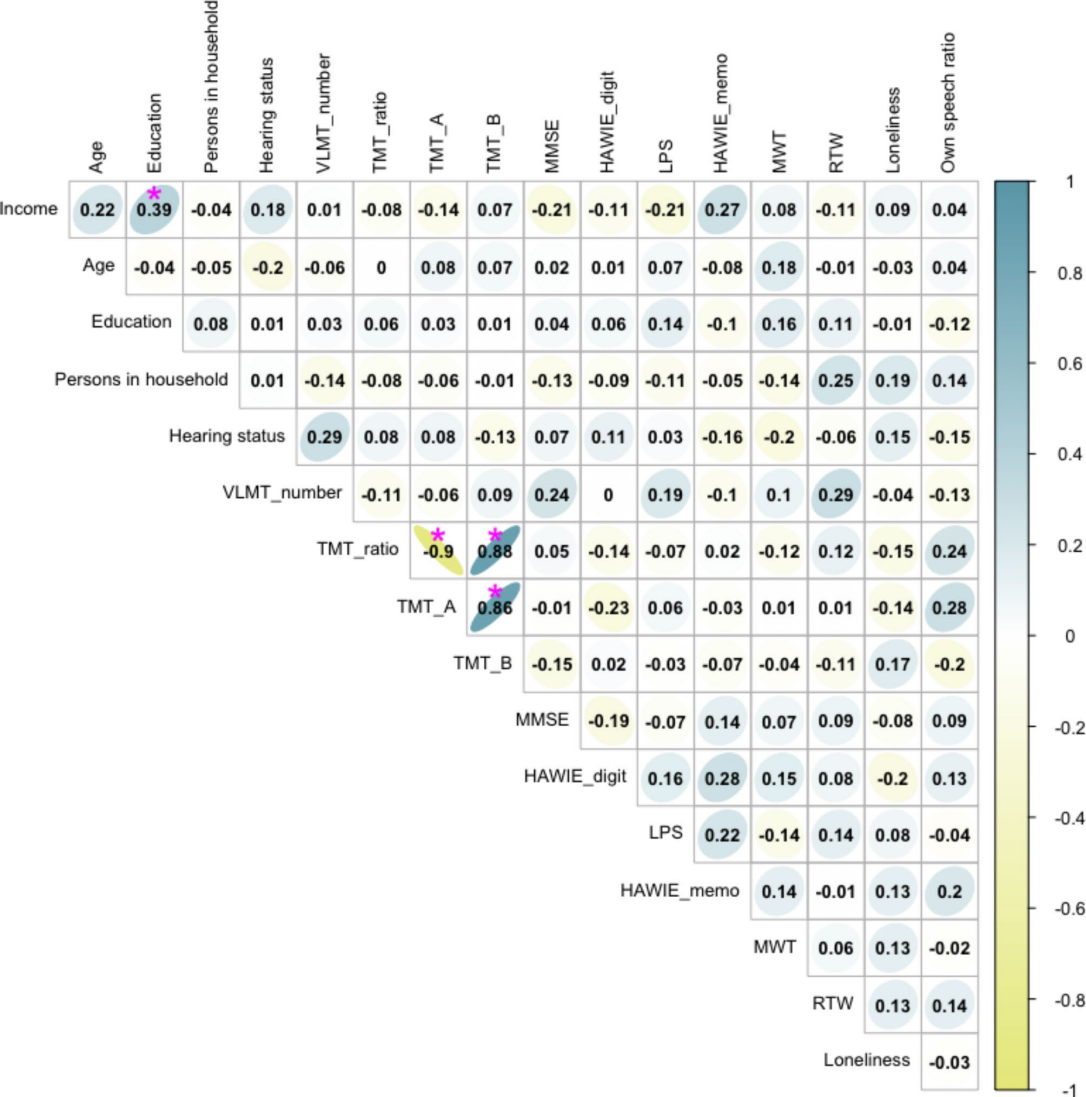
Partial Spearman correlation matrix of the model’s variable set. Significant bivariate correlations are highlighted by a magenta asterisk (FDR-adjusted for multiple comparisons). HAWIE_digit = Hamburg-Wechsler Intelligence Test for Adults digit-symbol test. HAWIE_memo = Hamburg-Wechsler Intelligence Test for Adults repeated numbers task. LPS = Leistungsprüfsystem (phonemic fluency). MMSE = Mini-Mental State Examination. MWT = Multiple Choice-Vocabulary-Intelligence Test (verbal abilities, semantic memory). RTW = Regensburger Wortflüssigkeitstest (semantic fluency). TMT_A = Trail Making Test Part A (numeric). TMT_B = Trail Making Test Part B (alphanumeric). TMT_r = Trail Making Test Ratio (B/A). VLMT_number = Verbal Learning and Memory Test, episodic memory.

In the descriptive, exploratory correlation network analysis (see Fig. 3), two major clusters emerged: one primarily encompassing cognitive ability variables, age, and subjective hearing status, and the second largely consisting of sociodemographic variables. Notably, the dependent variable from the elastic net regression, length of own speech, did not demonstrate strong bivariate correlations with the other included variables. The same is true for social loneliness, which was included to control for general social interaction. Furthermore, age exhibited an expected correlation with the TMT scores, but its correlation with other variables, including the length of own speech or subjective hearing status was less pronounced. For further reference, partial correlation coefficients including significance levels are provided in a correlation matrix plot (Fig. 4).

## Discussion

With a rapidly increasing aging population, cognitive aging researchers are exploring the impact of lifestyle factors for various health outcomes, including cognitive health, as these factors may provide cues for efficient interventions to maintain cognitive skills in late life or prevent cognitive decline. Of these lifestyle factors, social activity is of particular interest, as improving social relationships may offer a relatively accessible and cost-effective strategy to enhance cognitive functioning. Our study investigated, for the first time, an unobtrusively obtained real-life language indicator, that is the length of older adults’ real-life speech, as an indicator of social activity and its association with various cognitive abilities. Quantifying the length of older adults’ real-life speech using advanced technologies, such as the ones adopted in the current study allowing for unobtrusive collection of sensitive data protecting privacy of participants and related individuals, seems to be a feasible and efficient strategy to gain insights into adults’ amount of social activity in everyday life.

### Real-life speech length relates to cognitive functioning in distinct ways

As we have taken an exploratory approach, we openly investigated the relation of older adults’ length of speech to various sociodemographic and key cognitive ability variables. The chosen statistical modeling method (i.e., Elastic Net regression) is highly suited to this approach as it allows for assumption-free exploration of complex variable sets as well as for state-of-the-art control of possible multicollinearity. In terms of cognitive abilities, we found significant effects with working memory, processing speed and semantic fluency. That is, older adults with higher levels of working memory, processing speed and semantic fluency capacity spoke more in everyday life. Working memory and processing speed findings are in line with Kelly and colleagues’ extensive review^7^: These two cognitive abilities must be particularly crucial for engaging in conversations with others in everyday life moments. To be able to communicate efficiently, one must process interlocutors’ spoken language on the spot, maintain all relevant information in working memory and then respond timely to keep the conversation going (e.g.,^50,51^).

In contrast to Zuelsdorff and colleagues’ findings^29^, we did not find a positive relation with verbal learning and memory. This might be due to the difference in the way we measured “verbal interactions”. They have used a global, retrospective self-report, which has correlated significantly with their verbal learning and memory measure, the Rey Auditory Verbal Learning Test (RAVLT). This is a neuropsychological assessment designed as a list-learning paradigm in which the participant hears a list of 15 nouns and recalls as many words from the list as possible. Participants’ performance in this test was positively associated with their beliefs on how much they engaged in verbal interactions with others in a certain period of time. Our real-life speech length measure was not associated with that same measure of episodic memory nor with verbal knowledge, in line with Kelly and colleagues’ findings^7^: How much one speaks in real life does not seem to depend on how strong their episodic memory is or how wide their semantic knowledge (e.g., vocabulary) is.

How much one speaks, however, does seem to be related to verbal semantic fluency. This is in contrast to Kelly and colleagues’ findings^7^. Given the current study covers novel ground in exploring own speech length obtained from naturally produced and recorded speech utterances in relation to cognitive ability indicators, we can only speculate about the reason for this difference. For now, we assume that higher semantic fluency may motivate individuals to more actively engage in conversations or produce more of their own speech in general. Future research is clearly needed to replicate and finetune the relations between everyday speech and interindividual differences in cognition.

Different plausible mechanisms through which social relationships might contribute to cognitive functioning have been advanced in the literature (for an overview, see^8^). One possibility is that social interactions stimulate brain networks^52,53^: Social activities challenge older adults to participate in sophisticated interactions and conversations, which could maintain and enhance efficient neural networks^54^. This could be achieved through the building of a cognitive reserve capacity that buffers the brain against the risk of cognitive decline (e.g.,^55^). Social activity could also provide meaningful social tasks and roles, as well as a sense of purpose in late life^9^, which could have a direct impact on the brain, including limiting the stress response (e.g.,^56^). Investigating these mechanisms was not within the scope of the current study, however it is important to note that the interplay between language production and cognitive abilities is a complex relationship (e.g.,^4^).

### Real-Life speech length relates to differences in sociodemographic and subjective hearing status

In terms of sociodemographic and hearing correlates, older adults without a partner produced less real-life speech, which is a plausible and validating finding as to the usefulness of our length of own speech indicator for social activity. Demiray and Luo^22,24,25^ have used the EAR method to examine the linguistic qualities of older adults’ real-life speech across various interlocutors. They show, across studies, that the spouse is a key socializing partner for older adults in everyday life. For example, Demiray and colleagues^32^ examined reminiscence behavior in real-life conversations and showed that the spouse was a major reminiscence partner with important identity functions associated with this shared activity. Luo and colleagues^24^ found that older adults produced equally complex grammar as young adults did, when talking with their significant other. Luo and colleagues^22^ examined three linguistic measures that are associated with age-related cognitive changes: use of unique words, use of uncommon words, and grammatical complexity. They found no age effects on these linguistic measures when interlocutors were not taken into account. They showed that interlocutors influenced the production of unique words and grammatical complexity: Compared to talking with their spouse, participants used fewer unique words with children and friends; and used simpler grammatical structures with children, strangers, and in multiparty conversations. In sum, the spouse is a key social partner in late life, and the strongest predictor of how much older adults speak in everyday life.

The second strongest predictor in our analysis was subjective hearing status. We found that those who have higher hearing capacity spoke less. As puzzling as this finding may seem at first sight, intact hearing is required to engage in a conversation that involves both speaking and listening episodes (e.g.,^57^). Individuals with lower subjective hearing ability may engage in more one-sided conversations in which they are the primary speakers and leave less room for their conversation partners to also engage more actively. In fact, previous research has demonstrated that older adults with hearing loss often experience breakdown in communication. This results in withdrawal from social interactions in order to avoid difficult or embarrassing experiences; thereby, disrupting normal social behavior (e.g.,^58^). Recent systematic reviews confirm this link between hearing loss and increased social isolation and loneliness, emphasizing the relevance of hearing loss in healthy cognitive aging^57,59^.

We did not find a significant age effect on the length of real-life speech. That is, in our sample of community-dwelling and healthy older adults between ages 65 and 91, age was not a significant predictor of how much they spoke in the regression model and did not correlate significantly with any other variable on an exploratory level (see Figs. 3 and 4 for more details). This may in fact be due to the relatively high level of overall functioning of our sample, with greater homogeneity and a large portion of our sample being in the so-called Third Age, making it difficult to find meaningful interindividual differences within our sample of older adults. Future studies might benefit from a more balanced distribution across the full range of the older age lifespan, including a sufficient number of very old adults and those with more heterogenous health profiles in order to provide more detailed insights into the effects of aging on speech patterns and cognitive function.

### Strengths, limitations and future research

One strength of the current paper is that we obtained a full month of natural real-life speech recordings in a to date understudied population, namely community-dwelling older adults. On that basis, a second major strength of the present study is its identification and use of the length of a person’s own real-life speech as a proposed sensor-based, objective indicator of social activity, and to examine its relation with cognitive ability to complement existing theoretical notions and empirical findings about the interplay of social and cognitive functioning and the role of language in later life. A third significant strength is the combination of unobtrusive data sampling with ethically sound practices. The study ensured that the data collected were minimally invasive, thus capturing authentic behavior, while adhering to stringent ethical standards to protect the participants’ privacy and integrity. Through automated extraction processes, we minimized the risk of exposure and maintained the confidentiality of the data. Finally, most studies in the literature have focused on cognitive decline when examining the association between social activity and cognitive abilities in late life: Findings show that more socially active older adults experience less cognitive decline on average (e.g.,^5,8,18,52,60–62^). The contribution of the current study is its focus on healthy older adults and on examining this association within the context of healthy cognitive aging.

This study also has several limitations that need to be considered. First, there are issues concerning the temporal granularity of the Vocalise software, which limits the precision of our speech data extraction. The nature of our real-life data and current software limitations do not allow for a finer temporal resolution, which may affect the accuracy of our speech length measurements.

Second, the nature of individual speech data, including variations in the amount and timing of speech, as well as the randomness inherent in uncontrolled, real-world data sampling, could introduce biases into our dataset. The application of machine learning methods for speaker identification and extraction comes with its own set of challenges, and errors in this step could potentially affect our results. Given the novelty of this technology, many improvements can be expected in the near future including openly available and trainable models for research purposes.

Third, the data we recorded presented specific challenges. Issues such as background noise, multiple speakers, and the variability in recording conditions could all affect the accuracy of the speaker identification and speech extraction process. Furthermore, our quantitative approach does not allow for a qualitative investigation of older adults’ language production. That is, we have only measured the length of their speech with no focus on the tone, type or sentiment of the speech (e.g.,^63^). Future research could utilize automatic acoustic analyses to extract further qualitative information from the audio data about social activities (e.g.,^64^).

Moreover, automatic speech-to-text transcription is currently not possible due to the complex and noisy nature of our real-life audio data. The development of a language model for Swiss German, a dialect-rich language, for speech-to-text conversion is a future direction that might enhance the quality of our data. This would also allow for complex text analytics that can provide a window into older adults’ psychological and social worlds through their linguistic characteristics (e.g.,^32^).

Another limitation of our study is the absence of objective hearing assessments, such as pure-tone audiometry, which would provide a more comprehensive understanding of hearing status. Subjective self-report measures, while informative, may not fully capture the nuanced auditory capabilities addressed by objective tests and their interaction with cognitive as well as sociodemographic and health data (e.g.,^65^). Future research should incorporate both objective and more detailed subjective measures, such as the SSQ^66^, to better assess hearing performance across different domains.

The absence of refined self-report data, such as the questionnaire by Zuelsdorff and colleagues^17^, is a limitation of our study, as it could have complemented our objective measures of social engagement or activity. While we did assess social engagement with the UCLA Loneliness Scale, it did not yield significant results. Future research should incorporate detailed subjective questionnaires alongside objective measures to better evaluate the relationship between social engagement and cognitive functioning.

Excluding individuals with MMSE scores below 27 limits the range of cognitive performance in our study, potentially reducing variability needed to detect meaningful relationships between speech length and cognitive measures. This cut-off was chosen to avoid participants with any risk of pathological cognitive decline, but only a few individuals were excluded. The hereby introduced low variability in MMSE may have thus lowered the impact of any cognitive variable on the target variable, length of own speech. While the MMSE was selected to align with an ongoing longitudinal study in our group, future research should consider using the MoCA, which better detects cognitive heterogeneity and avoids ceiling effects^67^. All cognitive assessments were administered by trained research assistants in quiet, controlled environments to ensure appropriate testing conditions.

Although participants were aware they were being recorded, they were not informed of the exact timing, which likely minimized potential bias in their natural communication behavior. Furthermore, the method is well-accepted and compliance high as shown in many studies over the last 20 years (e.g.,^68,69^).

While the study did not focus on clinical applications, we acknowledge the need for future research to explore whether shorter recording periods could yield reliable data, both for scientific and clinical purposes. From a statistical perspective, the study’s sample size and the split between training and testing data could also be potential limitations. Using a standard 70–30 split for the Elastic Net approach, a larger sample size might have provided more robust and generalizable results. There is also a possibility of selection bias in the predictors, which could affect the validity of our findings. However, the robustness of our findings and the stability of our models, despite these potential limitations, speak to the validity and reliability of our data.

Future research addressing the issues mentioned above could contribute to a more refined understanding of the relationship between speech patterns and cognitive decline in the older adult population. One advancement in research methodology that we embrace is a multi-method approach in which the EAR is supported with other mobile sensing techniques, such as collecting additional sensory data (e.g., GPS) and momentary self-report (to collect unobservable information that cannot be captured by the EAR). The MOASIS project^34^ includes such rich ambulatory assessment data. In addition, a logical and important next step will be to complement our present between-person correlational findings with an analysis at the within-person level, in which we obtain daily indicators of own speech and link it to daily working memory, also examining cross-level interactions with other time-varying and person-level variables. In addition, the combination of long-term longitudinal trajectory data on cognitive ability maintenance versus change with real-life speech frequency will help determine the lead-lag relationship between verbal (i.e., social) activity and cognitive aging.

## Conclusion

Recently, researchers have been emphasizing that studies examining naturalistic speech are very rare^70,71^. In this study, we demonstrate how the EAR method can be used in novel ways to create both individual- and group-level corpora of spoken language, and in turn introduce a novel indicator of objectively sampled everyday social activity. Such data obtained from naturalistic observations are valuable for gaining insight into both social and cognitive processes in the daily lives of older adults.

Longer periods of social exchange with others might lead to better cognitive ability outcomes. However, it may also be that older adults who have stronger cognitive abilities speak more with others in daily life. In fact, intact working memory functioning and processing speed are crucial to both passively and actively follow and engage in a conversation. It is beyond the correlational design of this first exploratory study to determine the directionality of the speech-cognition relations. We believe, however, that the method introduced in this paper, both in terms of data acquisition and processing, lends itself to a wide range of future research to explore both the between-person and within-person associations between language, social activity, and cognitive functioning.

## Data Availability

Source data (audio recordings) are subject to legal restrictions and cannot be shared at this point due to data privacy issues. Aggregated data may be shared upon reasonable request to the authors (christina.roecke@uzh.ch).
